# In Favor of Establishment: Regulation of Chromatid Cohesion in Plants

**DOI:** 10.3389/fpls.2017.00846

**Published:** 2017-05-23

**Authors:** Pablo Bolaños-Villegas, Kuntal De, Mónica Pradillo, Desheng Liu, Christopher A. Makaroff

**Affiliations:** ^1^Laboratory of Molecular and Cell Biology, Fabio Baudrit Agricultural Research Station, University of Costa RicaAlajuela, Costa Rica; ^2^Department of Radiation Oncology, James Cancer Hospital and Comprehensive Cancer Center, The Ohio State University Wexner School of Medicine, ColumbusOH, United States; ^3^Departamento de Genética, Facultad de Biología, Universidad Complutense de MadridMadrid, Spain; ^4^Hughes Laboratories, Department of Chemistry and Biochemistry, Miami University, OxfordOH, United States

**Keywords:** cell division, CTF7, WAPL, PDS5, transposons, meiosis, recombination, DNA repair

## Abstract

In eukaryotic organisms, the correct regulation of sister chromatid cohesion, whereby sister chromatids are paired and held together, is essential for accurate segregation of the sister chromatids and homologous chromosomes into daughter cells during mitosis and meiosis, respectively. Sister chromatid cohesion requires a cohesin complex comprised of structural maintenance of chromosome adenosine triphosphatases and accessory proteins that regulate the association of the complex with chromosomes or that are involved in the establishment or release of cohesion. The cohesin complex also plays important roles in the repair of DNA double-strand breaks, regulation of gene expression and chromosome condensation. In this review, we summarize progress in understanding cohesion dynamics in plants, with the aim of uncovering differences at specific stages. We also highlight dissimilarities between plants and other eukaryotes with respect to the key players involved in the achievement of cohesion, pointing out areas that require further study.

## Introduction

In eukaryotes, DNA faithfully duplicates during the S phase of the cell cycle to produce sister chromatids. The newly duplicated sister chromatids are then tethered and held together by the cohesin complex until they segregate into new daughter cells (Uhlmann and Nasmyth, 1998). The cohesin complex is also involved in the repair of DNA double-strand breaks (DSBs), the regulation of gene expression and chromosome condensation (Guacci et al., 1997; Sjögren and Nasmyth, 2001; Onn et al., 2008; Nasmyth and Haering, 2009; Yuan et al., 2011; Lopez-Serra et al., 2013; Mehta et al., 2013; da Costa-Nunes et al., 2014).

**FIGURE 1:**
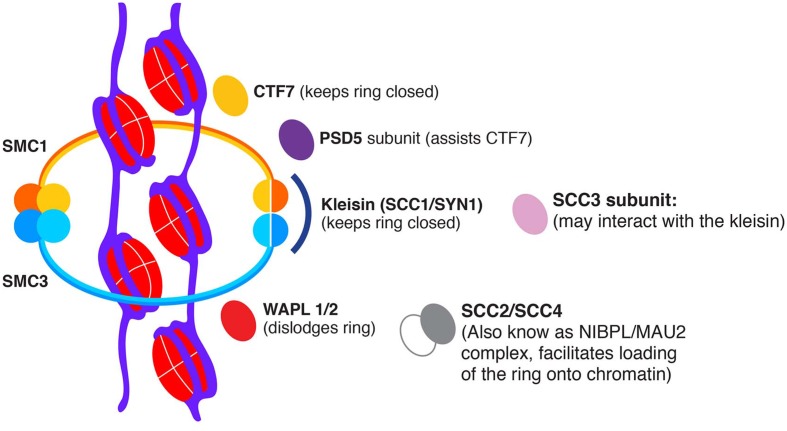
**Putative model of a plant cohesin ring.** The presumptive Arabidopsis SMC1/SMC3 cohesin complex may tether sister chromatids, and its activity may be regulated positively by CTF7 and negatively by WAPL. Other regulators are the kleisin SCC1, the subunit SCC3, cohesin regulator PDS5 and SCC2/SCC4.

In eukaryotic organisms, the cohesin core complex comprises four structural proteins: two structural maintenance of chromosome (SMC) adenosine triphosphatases (ATPases), SMC1 and SMC3; the α-kleisin sister chromatid cohesion protein 1 (SCC1); and the SCC3 subunit. The interaction of cohesin with chromosomes is regulated by the genes *PRECOCIOUS DISSOCIATION OF SISTERS 5* (*PDS5*) and *WINGS APART-LIKE* (*WAPL*). In animal cells, Sororin helps promote the stable association of cohesin with chromatin (Peters and Nishiyama, 2012). Tripartite rings are formed via the association of the SMC1-SMC3 heterodimer with SCC1 (Anderson et al., 2002; Haering et al., 2002). Different models have been proposed to explain the functional interaction of the complex with chromatin. The simplest model suggests that the ring entraps the two sister chromatids (Haering et al., 2008) (**Figure 1**). In other models, the interaction between different cohesin complexes can promote sister chromatid tethering (Eng et al., 2015). SCC1 also directly or indirectly associates with SCC3, PDS5, and WAPL (Haering et al., 2002).

Other SMC complexes exist, such as the condensin (SMC2/4) and SMC5/6 complexes, and their function is required in different contexts (Hirano, 2006). Condensins are multisubunit protein complexes that play a crucial role in the structural and functional organization of chromosomes (Ono et al., 2003). The majority of eukaryotes, including Drosophila possess two condensin complexes that participate in gene regulation, DNA repair and cell fate determination (Ono et al., 2003; Klebanow et al., 2016), while in Arabidopsis the condensin II complex is also important for conferring tolerance to excess soil Boron (Sakamoto et al., 2011). In addition to the SMC2/4 subunits, each complex may feature three non-SMC subunits, namely CAP-D2, CAP-G, and CAP-H for condensin I and CAP-D3, CAP-G2, and CAP-H2 for condensin II (Hirano, 2012). Arabidopsis CAP-D2 and CAP-D3 are required for pollen fertility and for preventing the association of centromeric repeats (Schubert et al., 2013). The eukaryotic SMC5/6 complex is primarily involved in DNA repair, replication fork stability, and possibly in the control of DNA topology (Verver et al., 2016). It consists of two SMC proteins and several non-SMC proteins (Andrews et al., 2005), which may interact with the ATPase head domain of SMC5 and SMC6 (Pebernard et al., 2004; Palecek et al., 2006). In Arabidopsis, the SMC5/SMC6 subunit AtMMS21 has been shown to regulate maintenance of root stem cells during embryogenesis and postembryonic stages (Xu et al., 2013). Given the evolutionary conservation of these complexes, it seems that their spatial organization and topology are very important to define their functionality (Gligoris and Löwe, 2016).

In this review, we summarize recent progress in understanding cohesion dynamics in plants, highlight differences at specific stages, key points of divergence between plant cohesin complexes and those from yeast and metazoans, and point out areas that require further study.

## The Core Cohesin Complex

In Arabidopsis, cohesion is mediated by the cohesin complex, consisting of two subunits of the SMC protein family, SMC1 and SMC3 (Liu et al., 2002). Both SMC1 and SMC3 are present in the Arabidopsis genome as single-copy genes (Liu et al., 2002). SMC1 and SMC3 are highly conserved among plant species and share the same characteristics: an N-terminal ATP binding domain, two large antiparallel coiled-coil regions separated by a hinge region, and a C-terminal DA box (Liu et al., 2002). The homozygous T-DNA knockouts of *SMC1* (*titan8-1* and *titan8-2*) and *SMC3* (*titan7-1 and titan7-2*) show developmental defects in both embryo and endosperm that result in an early arrest in seed development (Liu et al., 2002).

**FIGURE 2:**
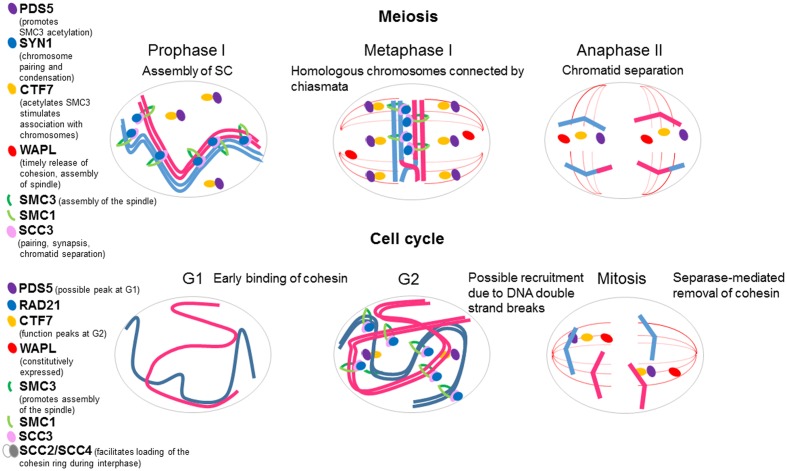
**Schematic representation of plant cohesin dynamics during mitosis and meiosis.** Cohesin complexes contribute to meiotic chromosome dynamics, since they influence on pairing (alignment of homologous chromosomes), synaptonemal complex (SC) formation (intimate association of homologous chromosomes), and recombination (DNA exchanges, reciprocal or not, between homologous sequences). During prophase I, sister chromatid cohesion and reciprocal exchanges (crossovers, COs) maintain the homologous chromosomes connected as a bivalent after the SC is disassembled. Afterward, cohesion is removed in two steps: from chromosome arms during first meiotic division and from centromeres during second meiotic division (top). During mitosis, the complexes might be involved in replication and segregation of chromatids (bottom).

Structural maintenance of chromosome 3 is found in both the cytoplasm and nucleus, bound to the nuclear matrix of somatic cells and in meiocytes (Lam et al., 2005). Specifically, it is localized from interphase to anaphase during mitosis, from premetiotic G_2_ to anaphase I during meiosis I, and in metaphase II centromeres during meiosis II (**Figure 2**). Strikingly, the protein is also present in the mitotic and meiotic spindle, so SMC3 may have additional roles in plant cells other than sister chromatid cohesion (Lam et al., 2005). No reports of Arabidopsis SMC1 have been published. In tomato, this protein, as well as SMC3, localizes along the axial elements (AEs), the precursors of the lateral elements (LEs) of the synaptonemal complex (SC), the tripartite structure that links homologous chromosomes in zygotene-pachytene meiocytes; but whether it is present in the cytoplasm and localizes to the spindle is unclear because studies were not conducted on whole cell mounts (Lhuissier et al., 2007).

Four orthologs of the kleisin subunit SCC1/radiation sensitive 21 (RAD21) have been detected in Arabidopsis and rice, and several *SCC1/RAD21* genes are present in other plant species (Golubovskaya et al., 2006). During the first meiotic division, the mitotic kleisin subunit SCC1 is replaced by RECOMBINATION 8 (REC8), (Klein et al., 1999) which plays a role during meiosis that SCC1/RAD21 cannot support (Watanabe and Nurse, 1999). In maize, the ortholog of *REC8* is *ABSENCE OF FIRST DIVISION 1* (*AFD1*) (Golubovskaya et al., 2006). AFD1 is essential for the elongation of AEs and immunolocalization studies revealed that it localizes to the LEs of the SC. The AFD1 protein is also required for RAD51 distribution on the chromosomes and is also important for homologous chromosome pairing (Golubovskaya et al., 2006). In the rice genome, the putative *REC8* ortholog is thought to be *RAD21-4*. RNA knock-down of this gene resulted in multiple aberrations during male meiosis, which included severe chromosome condensation, precocious segregation of homologous chromosomes and chromosome fragmentation (Zhang et al., 2006).

In the Arabidopsis genome, the *REC8* ortholog is *SYN1/DIF1* (Peirson et al., 1997; Bai et al., 1999; Bhatt et al., 1999; Cai et al., 2003). T-DNA *syn1* mutants are sterile in both male and female gametophytes, but the protein is dispensable for somatic development; vegetative growth appears normal in the mutants (Bai et al., 1999; Bhatt et al., 1999; da Costa-Nunes et al., 2006). Male meiocytes show severe defects in sister chromatid cohesion, homologous chromosome pairing, and chromosome condensation that result in the fragmentation of chromosomes and formation of polyads (Peirson et al., 1997; Bai et al., 1999; Bhatt et al., 1999; Cai et al., 2003). Transmission electron microscopy of chromosomes in *syn1* meiocytes show short stretches of SC surrounded by condensed chromatin in late pachytene, which suggests that SYN1 is essential for SC formation (Zhao et al., 2006). In the *syn1* mutant, the recombination machinery is partially functional since some recombination spots were seen in chromosomes (Zhao et al., 2006). Immunolocalization studies showed that SYN1 first appears on meiotic chromosomes beginning in late interphase. SYN1 antibody labels the developing chromosome axes beginning at early leptotene and lines the chromosome axes of paired chromosomes (Cai et al., 2003). A large portion of SYN1 dissociates from the chromosome arms during diplotene and diakinesis and by metaphase I the signal is only associated with the centromeres. SYN1 signal is not typically detected at late metaphase I and early anaphase I. Similar to Arabidopsis SYN1, yeast REC8 dissociates from chromosome arms and by metaphase I the signal is localized only at centromeres (Klein et al., 1999; Watanabe and Nurse, 1999).

Arabidopsis has three other kleisin genes, *SYN2/AtRAD21.1*, *SYN3/AtRAD21.2*, and *SYN4/AtRAD21.3*, which are expressed throughout the plant (Dong et al., 2001; Jiang et al., 2007). SYN3 plays an important role in the nucleolus of both somatic and meiotic cells and is also indispensable for megagametogenesis (Jiang et al., 2007), while *SYN2/AtRAD21.1* and *SYN4/AtRAD21.3* play roles in DNA repair and may represent mitotic cohesins (Dong et al., 2001; da Costa-Nunes et al., 2006). Plants homozygous for mutations in *AtRAD21.1* and *AtRAD21.3* showed a decrease in sister chromatid alignment in somatic cells, suggesting that they may represent the mitotic cohesins (da Costa-Nunes et al., 2006). *AtRAD21.1* has been shown to play a critical role in recovery after DNA damage during seed imbibition before germination (da Costa-Nunes et al., 2006), whereas *AtRAD21.3* appears to play a role in somatic DNA DSB repair (da Costa-Nunes et al., 2014).

In contrast to mammals, *SCC3* is present as a single-copy gene in Arabidopsis. The corresponding protein is 1,098 amino acids long and exhibits 21% sequence identity and 40% sequence similarity to its yeast homolog. The transcript is expressed strongly in roots, mature leaves, buds, and plantlets (Cromer et al., 2013). In general, T-DNA insertional mutations of *SCC3* result in embryo lethality; however, a weak allele, *scc3-1*, hypothesized to express a truncated protein, confers both mitotic and meiotic defects and homozygous *scc3-1* plants are dwarf and sterile (Chelysheva et al., 2005). Microscopy analysis revealed few dividing cells in root tips as compared with the wild type (WT), and male meiotic chromosomes showed defects in chromosome condensation, chromosome pairing and synapsis and presented early sister chromatid separation. During meiosis, SCC3 appears to localize at the chromosome axes until anaphase I, but during mitosis it is present throughout the entire cell cycle. Moreover, SYN1 binds normally to meiotic chromosomes in *scc3-1* plants, but in *syn1-1* plants, SCC3 localizes incorrectly to meiotic chromosome axes (Chelysheva et al., 2005). Whether the two proteins interact is still unknown.

## Loading of the Cohesin Complex

Before DNA replication, SCC2 and SCC4 (also known as the NIBPL/MAU2 complex) mediate the recruitment of cohesin to chromosomes in *Saccharomyces cerevisiae*, *Caenorhabditis elegans*, and humans (Ciosk et al., 2000; Gillespie and Hirano, 2004; Watrin et al., 2006; Onn et al., 2008; Nasmyth and Haering, 2009). However, these proteins are not required to maintain cohesion after the completion of DNA replication. In fact, large-scale mapping in several organisms such as *S. cerevisiae* and *Schizosaccharomyces pombe* has demonstrated that the cohesin complex and SCC2 bind non-randomly to chromosomes and that the respective binding loci may not overlap (Blat and Kleckner, 1999; Glynn et al., 2004; Lengronne et al., 2004; Weber et al., 2004; D’Ambrosio et al., 2008; Schmidt et al., 2009). Cohesin is enriched in regions around centromeres and at sites of convergent transcription, whereas SCC2 and SCC4 localize with transfer RNA genes (Glynn et al., 2004; D’Ambrosio et al., 2008). Cohesin may first associate with SCC2/SCC4, then relocate to sites of convergent transcription through the action of RNA polymerases (Lengronne et al., 2004; Hu et al., 2011; Fernius et al., 2013). The specific function of SCC2/SCC4 during loading of the cohesin complexes is unclear, but they may activate or prime the ATPase activity of SMC proteins, somehow allowing cohesin rings to entrap chromosomes (Haering et al., 2002; Arumugam et al., 2003; Gruber et al., 2003, 2006; Seitan et al., 2006). This behavior has been inferred from mutant SMC1 or SMC3 proteins that cannot hydrolyze ATP. These mutations lead to a phenotype that resembles that of *scc2* or *scc4* mutants in which cohesin rings are formed but fail to associate with chromosomes (Arumugam et al., 2003). Alternatively, it has been proposed that SCC2/SCC4 subunits might have a role in the remodeling of chromatin to facilitate the binding of cohesin (Hakimi et al., 2002; Huang et al., 2004; Ritchie et al., 2008).

In addition to SCC2/SCC4, other factors are required for the association of cohesin with chromosomes. For example, in *Xenopus* egg extracts, the CDC7/DRF1 kinase (DDK), a component of pre-replication complexes (pre-RCs), is essential for loading both SCC2/SCC4 and cohesin onto chromatin (Gillespie and Hirano, 2004; Takahashi et al., 2004, 2008; Ström et al., 2007). However, SCC2/SCC4 complexes have not been found associated with pre-RCs in yeast (Uhlmann and Nasmyth, 1998). In some instances, the kinetochore, transfer RNA transcription factors or proteins related to epigenetic mechanisms also participate in SCC2/SCC4-mediated loading of cohesin (Nonaka et al., 2002; Weber et al., 2004; D’Ambrosio et al., 2008). Nonetheless, although SCC2 and SCC4 are essential for cohesin loading, they are dispensable for cohesin maintenance and resolution during the S and G_2_ stages (Ciosk et al., 2000; Lengronne et al., 2006).

Functional characterization indicated that SCC2/SCC4 is essential for establishing sister chromatid cohesion in Arabidopsis (Sebastian et al., 2009). T-DNA insertional mutations in SCC2 and SCC4 lead to defects in embryo and endosperm development (Sebastian et al., 2009). Additionally, RNAi knockdown of *SCC2* leads to defects during male and female meiosis, including chromosome clumping, chromosome fragmentation, loss of chromatid cohesion, SCC3 mis-distribution and defects in segregation (Sebastian et al., 2009). The predicted protein sequence of Arabidopsis *SCC2* reveals a putative plant homeodomain (PHD) finger, a domain involved in chromatin organization and regulation of gene expression (Sebastian et al., 2009). Cytological analyses of T-DNA insertional lines *Atscc2-2*, *Atscc2-3*, *Atscc4-1* and *Atscc4-1* indicates that in these lines 25% of all embryos develop only up to the heart stage and show loss of bilateral symmetry, cell over-proliferation in the suspensor, and in the case of *Atscc2-2*, over-proliferation of the endosperm (Minina et al., 2017). In *Atscc4-1* and *Atssc2-2* analysis of the distribution of the auxin-response reporter *DR5rev::3xVENUS-N7* indicates that most of the reporter is confined to the basal cells of the suspensor, which is the opposite to the wild type, suggesting the existence of: (a) alterations in the embryogenic potential of the suspensor in the mutants, and (b) a role for both SCC4 and SCC2 in embryonic cell fate determination (Minina et al., 2017). It was also found that SCC4 interacts stably with the N-terminus of SCC2 *in planta* and in baker’s yeast, but this interaction is not required for proper localization of SCC4 to the plant nucleus in *Atscc2-2* (Minina et al., 2017). The authors interpreted both this finding and the colocalization of SCC4 with mitotic kleisin *RAD21.3/SYN4* during interphase as an indication that Arabidopsis SCC4 may play a special role in the determination of sites for cohesin loading on chromatin (Minina et al., 2017). Taken together all these results indicate that in Arabidopsis the SCC2 protein plays an important role during meiosis (Sebastian et al., 2009), while the SCC2/SCC4 complex regulates embryo and endosperm development with additional functions that are specific for each subunit (Minina et al., 2017).

## Cohesion Establishment and Maintenance

In yeast, sister chromatid cohesion is established during the S phase of the cell cycle by the activity of Establishment of cohesion 1/Chromosome transmission fidelity 7 (Eco1/Ctf7) acetyltransferase soon after cohesins are recruited to chromosomes (Skibbens et al., 1999; Tóth et al., 1999; Onn et al., 2008; Nasmyth and Haering, 2009; Yuan et al., 2011). Eco1/Ctf7 acetylates lysine residues in the Smc3 subunit, close to its ATPase domain (K112 and K113). These residues are highly conserved among eukaryotes and are also acetylated in human cells by two proteins, establishment of sister chromatid cohesion *N*-acetyltransferase 1 (ESCO1) and ESCO2 (Hou and Zou, 2005; Zhang et al., 2008). Then the acetylated Smc3 protein interacts stably with Scc1 and counteracts the activity of the Rad61/Wpl1 (Wapl) complex, which is thought to promote the disassociation of cohesin from chromosomes (Rolef Ben-Shahar et al., 2008; Unal et al., 2008; Zhang et al., 2008; Rowland et al., 2009; Woo et al., 2009), possibly by interacting directly with the Ser/Thr phosphatase PP4, which has been shown to target kleisin Rad21 for dephosphorylation (Birot et al., 2017).

Vertebrates express an additional essential cohesion regulator called Sororin (Rankin et al., 2005). This protein associates with cohesin via acetylation and antagonizes WAPL by binding to PDS5 (Nishiyama et al., 2010). PDS5 also promotes SMC3 acetylation (Vaur et al., 2012; Chan et al., 2013). Hence, it integrates an anti-establishment action (by WAPL) with its requirement for cohesion maintenance during the cell cycle progression (Schmitz et al., 2007; Nishiyama et al., 2010). The Arabidopsis genome contains five putative *PDS5* homologs that share similarity with fungal and mammal sequences (Mercier et al., 2001; Pradillo et al., 2015). Compromised expression of several *PDS5* genes leads to a significant reduction in seed production (Pradillo et al., 2015). Depletion of PDS5 proteins alters only slightly meiotic division but alters DNA repair by homologous recombination (HR) (Pradillo et al., 2015).

The establishment of cohesion occurs concomitantly with DNA replication (Uhlmann and Nasmyth, 1998). In addition to Eco1 acetyltransferase, other proteins, related to DNA replication, contribute to the establishment of the SCC. In yeast, Eco1/Ctf7 interacts with DNA replication factors such as proliferating cell nuclear antigen (PCNA, a DNA polymerase processivity factor) (Moldovan et al., 2006), replication factor C (a component of the clamp loader replication factor C) (Mayer et al., 2001), the DNA helix itself (Mayer et al., 2004), and various clamp loader subunits (Petronczki et al., 2004). Inactivation or mutations in Eco1/Ctf7 lead to defects such as chromosome mis-organization, mis-distribution of the cohesin complex and activation of cell cycle checkpoints (Skibbens et al., 1999; Tóth et al., 1999; Milutinovich et al., 2007). Deletions or mutations in *Rad61/Wpl1, Pds5, Smc3* and *Scc3* may suppress the effect of deletions in *Eco1/Ctf7*, which suggests a degree of functional redundancy in the activity of Eco1/Ctf7 or that other factors can modify cohesin to counteract the activity of Eco1/Ctf7 during the establishment of cohesion (Warren et al., 2004; Rolef Ben-Shahar et al., 2008; Rowland et al., 2009; Sutani et al., 2009; Chen et al., 2012).

Experimental evidence indicates that Arabidopsis ECO1/CTF7 can functionally replace its yeast ortholog (Jiang et al., 2010; Bolaños-Villegas et al., 2013; Singh et al., 2013). Arabidopsis CTF7 lacks an N-terminal extension common in other organisms but features a PCNA-interacting protein (PIP) box, a C_2_H_2_ zinc finger motif and an acetyltransferase domain (Jiang et al., 2010). Similar to other species, Arabidopsis CTF7 appears to have a dosage-dependent function. Heterozygous *ctf7* plants exhibit defects in the development of female gametophytes, with no obvious defects in microsporogenesis. Vegetative growth is normal in these plants, but siliques contain fewer seeds than in WT plants and many show embryonic developmental defects. Inactivation of Arabidopsis CTF7 typically results in embryo lethality; however, homozygous *ctf7* mutant plants, which are completely sterile, can be obtained at very low frequencies. These plants show a more drastic phenotype: they are dwarf and feature fewer epidermal cells per area. Also, cell cycle progression is defective (Bolaños-Villegas et al., 2013). Furthermore, *ctf7* mutant plants exhibit a severe loss of sister chromatid cohesion during mitosis and meiosis as well as significantly reduced localization of cohesin onto chromosomes (Bolaños-Villegas et al., 2013). The absence of ECO1/CTF7 impairs cytosine methylation, especially CG methylation (Bolaños-Villegas and Jauh, 2015). In addition, genes involved in HR are upregulated, which suggests defects in DNA repair (Bolaños-Villegas et al., 2013). Similar phenotypes were observed in plants transformed with a dexamethasone-inducible *CTF7*-RNAi construct. Finally, overexpression of the *CTF7* genomic sequence leads to ovule arrest at female gametophyte 1 stage (Liu and Makaroff, 2015).

Extensive studies in different species have shown that WAPL controls mitotic sister chromatid cohesion and takes part in the removal of cohesin (Kueng et al., 2006). In Drosophila, WAPL has an important role in the organization of heterochromatin (Vernì et al., 2000). The *WAPL* genomic sequence features a conserved C-terminus that may be a determinant of cohesin and a divergent N-terminal domain that in humans contains a PDS5 binding domain (Chatterjee et al., 2013; Ouyang et al., 2013). Although the effect of inactivation of WAPL during mitosis has been studied in several organisms (Cunningham et al., 2012; Chatterjee et al., 2013; Ouyang et al., 2013), much less is known about its role during meiosis.

The Arabidopsis genome contains two *WAPL* genes that appear to have a significant role in the removal of cohesin in the prophase (De et al., 2014). Arabidopsis plants homozygous for either of the *wapl* mutations have no obvious phenotype, but double homozygous plants show reduced fertility and severe defects in male meiosis, including defective organization of heterochromatin regions during prophase I, altered pairing of homologous chromosomes and delayed cohesin release during the first meiotic division. Assembly of the meiotic spindle is also severely impaired in double mutants. These problems may lead to the formation of chromosome bridges, broken chromosomes, uneven segregation of chromosomes and aneuploid gametes (De et al., 2014). In contrast, cohesin complexes appear to be removed normally in somatic cells (De et al., 2014). Hence, Arabidopsis *WAPL* genes may play a critical role during meiosis, and mechanisms involved in the removal of cohesin during prophase may vary between mitosis and meiosis in plants. Additionally, inactivation of the two Arabidopsis *WAPL* genes can suppress the lethal phenotype produced by the lack of CTF7 (De et al., 2016) and allows for normal vegetative growth and production of a reduced number of viable seeds (De et al., 2016). Immunolocalization of SYN1 in meiocytes confirmed that the release of cohesin during diakinesis is recovered in *wapl1 wapl2 ctf7* triple homozygous mutant plants (De et al., 2016). However, comet assay experiments in vegetative tissues revealed that both WAPL1/2 and ECO1/CTF7 are important for the repair of DNA DSBs during the cell cycle in Arabidopsis (De et al., 2016). In addition, flow cytometry revealed a high level of aneuploidy in vegetative tissues of the triple mutant (De et al., 2016). All these results demonstrate that WAPL1/2 is important for the timely release of cohesion during meiosis and that inactivation of WAPL1/2 most-likely abrogates the requirement for SMC3 acetylation by CTF7 during mitosis (De et al., 2016). These plants are still able to develop and reproduce, which suggests the presence of an alternative cohesion pathway that awaits proper identification and functional characterization.

The Arabidopsis SMC-like gene *SWITCH* (*SWI1*), also known as *DYAD*, also plays a role in meiotic chromosome structure, maintenance and cohesion. The name is due to its function as a master controller of the switch from mitosis to meiosis (Mercier et al., 2001; Schubert, 2009). The corresponding mutant exhibits 10 univalents (instead of 5 bivalents) at the end of prophase I. Subsequently, chromatids lose their cohesion and their appear individually at metaphase I (Mercier et al., 2001, 2003). *SWI1* is a plant-specific gene that has been characterized in several species. The maize homolog is *AMEIOTIC1* (*AM1*). In *am1* mutants premeiotic cells undergo mitosis instead of meiosis and meiotic-specific cohesins are not installed on chromosomes (Pawlowski et al., 2009). In rice, OsAM1 is required for meiotic progression and the mutant fails to load OsREC8 on chromosome axes (Che et al., 2011).

Cohesion establishment and maintenance is also controlled by several posttranslational modifications. In addition to the acetylation mentioned previously, phosphorylation and SUMOylation play an essential role during cohesion establishment. Indeed, cohesin SUMOylation is indispensable for the entrapment of sister chromatids (Almedawar et al., 2012). In this sense, SUMO accumulates at DNA damage sites in S/G_2_-phase human cells in a cohesin-dependent manner. This modification affects SCC1 and promotes DNA repair by sister chromatid exchange by antagonizing WAPL (Wu et al., 2012).

## Cohesin Dissociation

During cell division, cohesin needs to be removed for segregation of sister chromatids. The dissociation of cohesin is tightly regulated and takes place during two phases that involve different factors (Sumara et al., 2000; Waizenegger et al., 2000; Peters and Nishiyama, 2012). During the mitotic prophase and prometaphase stages, most cohesins are removed from chromosome arms, but those at the centromere stay. However, shortly before the onset of mitotic anaphase, all remaining chromosome-bound cohesin (mainly at centromeres) is removed when SCC1 is cleaved by Separase (Uhlmann et al., 1999; Sumara et al., 2000; Tomonaga et al., 2000; Waizenegger et al., 2000; Losada et al., 2002).

In vertebrates, Sororin is targeted for phosphorylation by cyclin-dependent kinase 1 (CDK1)/Cyclin B, which facilitates the action of PDS5–WAPL. This complex takes part in the release of cohesin from the chromosome arms (Schmitz et al., 2007; Nishiyama et al., 2010). The fraction of cohesin that remains at centromeres is protected by Shugoshin 1 protein (SGO1), which mediates the recruitment of Phosphatase 2A to protect cohesin against phosphorylation and hinder its release (Kitajima et al., 2004; Tang et al., 2006; Rivera and Losada, 2009). Other proteins such as Haspin (a histone H3 kinase) and Prohibitin 2 have been found involved in the protection of cohesion at centromeres (Dai et al., 2006).

The bi-orientation of chromosomes at metaphase is possible because cohesin is preserved at centromeres. Before metaphase, the anaphase-promoting complex (APC/C) remains inactive and Separase is inhibited by Securin and Cyclin B (Stemmann et al., 2001; Toyoda et al., 2002; Musacchio and Salmon, 2007). This pathway is regulated by the spindle assembly checkpoint (SAC). At the onset of anaphase, the SAC is disrupted and APC/C becomes active and targets Securin and Cyclin B for ubiquitylation and destruction (Uhlmann et al., 1999; Hauf et al., 2001; Musacchio and Salmon, 2007). Free from its inhibitors, Separase is released and activated (Nasmyth, 2000). At the same time, SGO1 is released from the centromere and SCC1 is phosphorylated. Separase then proceeds to cleave SCC1 and remove cohesin from sister chromatids (Nasmyth, 2000; Hauf et al., 2001). Finally, SMC3 is deacetylated by the histone lysine deacetylase 1 for reuse in the next cycle (Rivera and Losada, 2010).

During meiosis, cleavage along chromosome arms is pivotal for disjunction of homologous chromosomes at anaphase I, but it must not occur at centromeres, because cohesion of sister chromatids is indispensable for their correct bi-orientation at metaphase II (Bai et al., 1999). REC8, and other meiotic-specific factors such as SMC1β (SMC1) or Stromal Antigen 3 (SCC3) work in concert to protect centromeric cohesion during anaphase I and interkinesis (Dong et al., 2001; Prieto et al., 2001; Revenkova et al., 2001).

In Arabidopsis, the protection of meiotic centromeric cohesion depends on several proteins including SGO1 and SGO2, which are required at anaphase I, and PATRONUS 1 (PANS1), which is required at interkinesis and meiosis II and is presumably targeted by the APC/C complex (Cromer et al., 2013; Zamariola et al., 2013). In *sgo1 sgo2* double mutant plants, immunolabeling for REC8/SYN1 suggested that this protein is not present at metaphase II (Cromer et al., 2013). The *pans1* mutant features up to 10 single chromatids at each metaphase II plate and no REC8/SYN1 signal at metaphase II in chromatids (Cromer et al., 2013). PANS1 may protect REC8 or may inhibit cohesin release by WAPL inactivation (Cromer et al., 2013; Zamariola et al., 2013).

The predicted Arabidopsis Separase protein (extra spindle pole bodies 1, ESP1) is significantly longer than the corresponding proteins from yeast, worm, and fly, but is similar to the mammalian protein (Liu and Makaroff, 2006). ESP1 proteins from different organisms show high similarity in the C-terminus, which features a C-50 peptidase domain (Liu and Makaroff, 2006). The ESP1 peptidase domain shares approximately 20% sequence identity with the mammalian enzyme. However, the Arabidopsis ESP1 peptidase domain is considerably longer than those found in other organisms (700 vs. ∼400–470 amino acids) (Liu and Makaroff, 2006). Moreover, this domain consists of a predicted 2Fe-2S-Ferredoxin domain that is not present in other organisms. ESP1 in Arabidopsis contains an EF-hand/calcium-binding domain, which is also present in budding yeast, where it is important for initiation or maintenance of its association with the spindle (Jensen et al., 2001; Liu and Makaroff, 2006). The calcium-binding domain may have the same function in plants, but this has not yet been proven. Analysis of T-DNA insertional mutants suggests that *ESP1* is an essential gene in that no homozygous plants from two different alleles could be obtained (Liu and Makaroff, 2006). Moreover 25% of the seeds from heterozygous plants for the T-DNA insertions showed enlarged endosperm nuclei and nucleoli, a failure of the endosperm to cellularize, and embryo arrest at the globular stage, indicating that the protein is essential for embryo development (Liu and Makaroff, 2006). The *radially swollen 4* (*rsw4*) mutant is a temperature-sensitive line that contains a mis-sense mutation in *ESP1* (Wu et al., 2010); replicated chromosomes fail to disjoin in roots. In addition, the roots of *rsw4* accumulate high levels of the mitotic-specific Cyclin B1 and show disorganized cortical microtubules. However, how inactivation of ESP1 specifically affects Cyclin B1 remains to be determined (Liu and Makaroff, 2006).

The role of ESP1 in Arabidopsis mitosis and meiosis has also been investigated by means of an RNAi construct driven by the *35S* and meiotic-specific *DMC1* promoters (Yang et al., 2009). The inability to recover RNAi plants containing the *35S* promoter suggested that *ESP1* is an essential gene during mitosis. RNAi plants containing the *DMC1* promoter showed entangled and stretched chromosomes during anaphases I and II (Yang et al., 2009). In addition, chromosome bridges and DNA fragmentation were observed, which suggested that *ESP1* is an essential gene for HR as well as chromosome segregation during both meiotic divisions (Liu and Makaroff, 2006; Yang et al., 2009). SYN1 and SMC3 signals persisted along the chromosome arms and the centromeres throughout meiosis in DMC1-*ESP1*-RNAi plants. *ESP1* RNAi knockdown during meiosis induced non-homologous association of centromeres, disruption of the radial microtubule system after telophase II, and disruption of the nuclear cytoplasm, which resulted in multinucleate microspores (Yang et al., 2009). Thus, ESP1 appears to function beyond the removal of cohesin in plant cells. Despite the importance of ESP1, little is known about the mechanism of its regulation in plants (Yang et al., 2009). Analysis of plant genomes has failed to identify a putative plant homolog of Securin, and no experimental work has been conducted on the activation of the Separase pathway.

## Role of Cohesin in Repair of DSBs

Cohesin is important for postreplicative repair of DSBs in both mitosis and meiosis (Klein et al., 1999; Cortés-Ledesma and Aguilera, 2006). The essential function of cohesin in DNA repair is to allow a DSB on one sister to be repaired using the undamaged sister as a template. Thus, it brings the two sister chromatids into close proximity to facilitate the repair by HR. In budding yeast, cohesin is removed from the chromatin at DSB sites to promote DSB resection and repair (McAleenan et al., 2013).

As discussed above, *SYN2/AtRAD21.1* and *SYN4/AtRAD21.3* have been shown to play roles in DNA repair (da Costa-Nunes et al., 2006, 2014). Further, results from the characterization of *ctf7* and *wapl1 wapl2 ctf7* single and triple mutants suggested that the failure to establish and regulate cohesion leads to the expression of genes involved in HR and the establishment of cohesion, including *ATM*, *BRCA1*, *RAD51*, *PARP2*, *SMC5, SMC6B*, and *TOPOII*-α in vegetative tissues (Bolaños-Villegas et al., 2013; De et al., 2016). The basis for these changes in gene expression is not well understood and whether alternate error-prone DNA repair mechanisms such as non-homologous end joining (NHEJ) are activated is unclear. In this context, it is worth mentioning that in human cells the cohesin complex contributes to the protection of distinct double-strand ends in the NHEJ DNA repair pathway, helping to avoid genome rearrangements in S/G_2_ phases (Gelot et al., 2016). It has been also demonstrated that the interaction between BREAST CANCER 2 (BRCA2) and cohesin via PDS5 is important for HR (Brough et al., 2012). In Arabidopsis, *PDS5* genes are overexpressed upon exposure to γ-rays. Furthermore, the absence of PDS5 proteins causes hypersensitivity to DNA damaging agents and severely reduced HR, which is probably related to reduced expression of *SMC6* genes (Pradillo et al., 2015). The Arabidopsis SMC5/SMC6 complex meliorates sister chromatid alignment after DNA damage, allowing DNA repair by HR (Mengiste et al., 1999; Watanabe et al., 2009). Conversely, the reduced repair efficiency by HR in *smc6b* mutants may facilitate gene editing (Qi et al., 2013). It has been proposed that cohesin and SMC5/SMC6 have partially overlapping functions and can complement one another if necessary (Tapia-Alveal et al., 2014).

Beyond its function in DNA segregation and repair, cohesin influences other important biological processes such as the regulation of gene expression, duplication of centrosomes and spindle polar bodies, and chromosome condensation (Mehta et al., 2013). In meiosis, cohesin complexes are also important for repairing DSBs. Unlike mitosis, during meiosis the formation of DSBs is programmed and most of them are repaired using non-sister chromatids as templates. Meiotic cohesin complexes also influence chromosome organization to ensure proper chromosome pairing, synapsis, and recombination (Bardhan, 2010; Zamariola et al., 2014). They also have a role in centromere coupling, a mechanism by which non-homologous centromeres pair during prophase I in HR-defective mutants (Tsubouchi and Roeder, 2005) and in bouquet formation, the clustering of telomeres anchored to the nuclear envelope at early meiotic stages (Golubovskaya et al., 2006; Storlazzi et al., 2008).

## Conclusion

The establishment of chromatid cohesion is crucial for ensuring accurate chromosome dynamics throughout the cell cycle. In plant cells, it is also essential for the development of embryos and seeds and the ability of plants to deal with DNA damage caused by ionizing radiation and faulty DNA replication. During meiosis, cohesin forms a platform for the assembly of the SC, plays an essential role in the exchange between homologous chromosomes and ensures their correct segregation at anaphase I. The regulation of meiotic and mitotic processes has a far-reaching effect on the survival and propagation of a species. Also, for agricultural applications, the study and characterization of genes involved in the establishment of cohesion has potential to enhance the long-term survival, reproduction and adaptation of crops under adverse environmental conditions, including increased UV radiation and the presence of genotoxic agents in soil and water. In addition, cohesin manipulation could be an useful tool to generate clonal seeds by apomixis, a type of asexual reproduction that avoids meiosis. Further work is needed to continue the characterization of plant cohesin complexes, the mechanics of its regulation and to explore its potential application for plant breeding.

## Author Contributions

All authors listed, have made substantial, direct and intellectual contribution to the work, and approved it for publication.

## Conflict of Interest Statement

The authors declare that the research was conducted in the absence of any commercial or financial relationships that could be construed as a potential conflict of interest. The reviewer MPAM and handling Editor declared their shared affiliation, and the handling Editor states that the process nevertheless met the standards of a fair and objective review.

## References

[B1] Almedawar S. Colomina N. Bermúdez-López M. Pociño-Merino I. Torres-Rosell J. (2012). A SUMO-dependent step during establishment of sister chromatid cohesion. *Curr. Biol.* 22 1576–1581. 10.1016/j.cub.2012.06.046 22771040

[B2] Anderson D. E. Losada A. Erickson H. P. Hirano T. (2002). Condensin and cohesin display different arm conformations with characteristic hinge angles. *J. Cell Biol.* 156 419–424. 10.1083/jcb.200111002 11815634 PMC2173330

[B3] Andrews E. A. Palecek J. Sergeant J. Taylor E. Lehmann A. R. Watts F. Z. (2005). Nse2, a component of the Smc5-6 complex, is a SUMO ligase required for the response to DNA damage. *Mol. Cell. Biol.* 25 185–196. 10.1128/MCB.25.1.185-196.2005 15601841 PMC538766

[B4] Arumugam P. Gruber S. Tanaka K. Haering C. H. Mechtler K. Nasmyth K. (2003). ATP hydrolysis Is required for cohesin’s association with chromosomes. *Curr. Biol.* 13 1941–1953. 10.1016/j.cub.2003.10.036 14614819

[B5] Bai X. Peirson B. N. Dong F. Xue C. Makaroff C. A. (1999). Isolation and characterization of SYN1, a RAD21-like gene essential for meiosis in Arabidopsis. *Plant Cell* 11 417–430. 10.1105/tpc.11.3.417 10072401 PMC144192

[B6] Bardhan A. (2010). Many functions of the meiotic cohesin. *Chromosom. Res.* 18 909–924. 10.1007/s10577-010-9169-0 21086039

[B7] Bhatt A. M. Lister C. Page T. Fransz P. Findlay K. Jones G. H. (1999). The DIF1 gene of Arabidopsis is required for meiotic chromosome segregation and belongs to the REC8/RAD21 cohesin gene family. *Plant J.* 19 463–472. 10.1046/j.1365-313X.1999.00548.x 10504568

[B8] Birot A. Eguienta K. Vazquez S. Claverol S. Bonneu M. Ekwall K. (2017). A second Wpl1 anti-cohesion pathway requires dephosphorylation of fission yeast kleisin Rad21 by PP4. *EMBO J.* 10.15252/embj.201696050 [Epub ahead of print]. PMC5430217 28438891

[B9] Blat Y. Kleckner N. (1999). Cohesins bind to preferential sites along yeast chromosome III, with differential regulation along arms versus the centric region. *Cell* 98 249–259. 10.1016/S0092-8674(00)81019-3 10428036

[B10] Bolaños-Villegas P. Jauh G.-Y. (2015). Reduced activity of Arabidopsis chromosome-cohesion regulator gene CTF7/ECO1 alters cytosine methylation status and retrotransposon expression. *Plant Signal. Behav.* 10:e1013794 10.1080/15592324.2015.1013794 PMC4622472 26039473

[B11] Bolaños-Villegas P. Yang X. Wang H.-J. Juan C.-T. Chuang M.-H. Makaroff C. A. (2013). Arabidopsis CHROMOSOME TRANSMISSION FIDELITY 7 (AtCTF7/ECO1) is required for DNA repair, mitosis and meiosis. *Plant J.* 75 927–940. 10.1111/tpj.12261 23750584 PMC3824207

[B12] Brough R. Bajrami I. Vatcheva R. Natrajan R. Reis-Filho J. S. Lord C. J. (2012). APRIN is a cell cycle specific BRCA2-interacting protein required for genome integrity and a predictor of outcome after chemotherapy in breast cancer. *EMBO J.* 31 1160–1176. 10.1038/emboj.2011.490 22293751 PMC3297997

[B13] Cai X. Dong F. Edelmann R. E. Makaroff C. A. (2003). The Arabidopsis SYN1 cohesin protein is required for sister chromatid arm cohesion and homologous chromosome pairing. *J. Cell Sci.* 116 2999–3007. 10.1242/jcs.00601 12783989

[B14] Chan K.-L. Gligoris T. Upcher W. Kato Y. Shirahige K. Nasmyth K. (2013). Pds5 promotes and protects cohesin acetylation. *Proc. Natl. Acad. Sci. U.S.A.* 110 13020–13025. 10.1073/pnas.1306900110 23878248 PMC3740900

[B15] Chatterjee A. Zakian S. Hu X.-W. Singleton M. R. (2013). Structural insights into the regulation of cohesion establishment by Wpl1. *EMBO J.* 32 677–687. 10.1038/emboj.2013.16 23395900 PMC3590988

[B16] Che L. Tang D. Wang K. Wang M. Zhu K. Yu H. (2011). OsAM1 is required for leptotene-zygotene transition in rice. *Cell Res.* 21 654–665. 10.1038/cr.2011.7 21221128 PMC3203663

[B17] Chelysheva L. Diallo S. Vezon D. Gendrot G. Vrielynck N. Belcram K. (2005). AtREC8 and AtSCC3 are essential to the monopolar orientation of the kinetochores during meiosis. *J. Cell Sci.* 118 4621–4632. 10.1242/jcs.02583 16176934

[B18] Chen Z. McCrosky S. Guo W. Li H. Gerton J. L. (2012). A genetic screen to discover pathways affecting cohesin function in *Schizosaccharomyces pombe* identifies chromatin effectors. *G3* 2 1161–1168. 10.1534/g3.112.003327 23050226 PMC3464108

[B19] Ciosk R. Shirayama M. Shevchenko A. Tanaka T. Toth A. Shevchenko A. (2000). Cohesin’s binding to chromosomes depends on a separate complex consisting of Scc2 and Scc4 proteins. *Mol. Cell* 5 243–254. 10.1016/S1097-2765(00)80420-7 10882066

[B20] Cortés-Ledesma F. Aguilera A. (2006). Double-strand breaks arising by replication through a nick are repaired by cohesin-dependent sister-chromatid exchange. *EMBO Rep.* 7 919–926. 10.1038/sj.embor.7400774 16888651 PMC1559660

[B21] Cromer L. Jolivet S. Horlow C. Chelysheva L. Heyman J. De Jaeger G. (2013). Centromeric cohesion is protected twice at meiosis, by SHUGOSHINs at anaphase i and by PATRONUS at interkinesis. *Curr. Biol.* 23 2090–2099. 10.1016/j.cub.2013.08.036 24206843

[B22] Cunningham M. D. Gause M. Cheng Y. Noyes A. Dorsett D. Kennison J. A. (2012). Wapl antagonizes cohesin binding and promotes Polycomb-group silencing in *Drosophila*. *Development* 139 4172–4179. 10.1242/dev.084566 23034634 PMC3478686

[B23] da Costa-Nunes J. A. Bhatt A. M. O’Shea S. West C. E. Bray C. M. Grossniklaus U. (2006). Characterization of the three *Arabidopsis thaliana* RAD21 cohesins reveals differential responses to ionizing radiation. *J. Exp. Bot.* 57 971–983. 10.1093/jxb/erj083 16488915

[B24] da Costa-Nunes J. A. Capitão C. Kozak J. Costa-Nunes P. Ducasa G. M. Pontes O. (2014). The AtRAD21.1 and AtRAD21.3 Arabidopsis cohesins play a synergistic role in somatic DNA double strand break damage repair. *BMC Plant Biol.* 14:353 10.1186/s12870-014-0353-9 PMC4273318 25511710

[B25] Dai J. Sullivan B. A. Higgins J. M. G. (2006). Regulation of mitotic chromosome cohesion by Haspin and Aurora B. *Dev. Cell* 11 741–750. 10.1016/j.devcel.2006.09.018 17084365

[B26] D’Ambrosio C. Schmidt C. K. Katou Y. Kelly G. Itoh T. Shirahige K. (2008). Identification of cis-acting sites for condensin loading onto budding yeast chromosomes. *Genes Dev.* 22 2215–2227. 10.1101/gad.1675708 18708580 PMC2518811

[B27] De K. Sterle L. Krueger L. Yang X. Makaroff C. A. (2014). *Arabidopsis thaliana* WAPL is essential for the prophase removal of cohesin during meiosis. *PLoS Genet.* 10:e1004497 10.1371/journal.pgen.1004497 PMC4102442 25033056

[B28] De K. Villegas P. B. Mitra S. Yang X. Homan G. Jauh G.-Y. (2016). The opposing actions of Arabidopsis CTF7 and WAPL1/2: differences in mitotic and meiotic cells. *Plant Cell* 28 521–536. 10.1105/tpc.15.00781 26813623 PMC4790872

[B29] Dong F. Cai X. Makaroff C. A. (2001). Cloning and characterization of two Arabidopsis genes that belong to the RAD21/REC8 family of chromosome cohesin proteins. *Gene* 271 99–108. 11410371 10.1016/s0378-1119(01)00499-1

[B30] Eng T. Guacci V. Koshland D. (2015). Interallelic complementation provides functional evidence for cohesin-cohesin interactions on DNA. *Mol. Biol. Cell* 26 4224–4235. 10.1091/mbc.E15-06-0331 26378250 PMC4642856

[B31] Fernius J. Nerusheva O. O. Galander S. Alves F. D. L. Rappsilber J. Marston A. L. (2013). Cohesin-dependent association of Scc2/4 with the centromere initiates pericentromeric cohesion establishment. *Curr. Biol.* 23 599–606. 10.1016/j.cub.2013.02.022 23499533 PMC3627958

[B32] Gelot C. Guirouilh-Barbat J. Le Guen T. Dardillac E. Chailleux C. Canitrot Y. (2016). The cohesin complex prevents the end joining of distant DNA double-strand ends. *Mol. Cell* 61 15–26. 10.1016/j.molcel.2015.11.002 26687679

[B33] Gillespie P. J. Hirano T. (2004). Scc2 couples replication licensing to sister chromatid cohesion in *Xenopus* egg extracts. *Curr. Biol.* 14 1598–1603. 10.1016/j.cub.2004.07.053 15341749

[B34] Gligoris T. Löwe J. (2016). Structural insights into ring formation of cohesin and related SMC complexes. *Trends Cell Biol.* 26 680–693. 10.1016/j.tcb.2016.04.002 27134029 PMC4989898

[B35] Glynn E. F. Megee P. C. Yu H. G. Mistrot C. Unal E. Koshland D. E. (2004). Genome-wide mapping of the cohesin complex in the yeast *Saccharomyces cerevisiae*. *PLoS Biol.* 2:e259 10.1371/journal.pbio.0020259 PMC490026 15309048

[B36] Golubovskaya I. N. Hamant O. Timofejeva L. Wang C.-J. R. Braun D. Meeley R. (2006). Alleles of afd1 dissect REC8 functions during meiotic prophase I. *J. Cell Sci.* 119 3306–3315. 10.1242/jcs.03054 16868028

[B37] Gruber S. Arumugam P. Katou Y. Kuglitsch D. Helmhart W. Shirahige K. (2006). Evidence that loading of cohesin onto chromosomes involves opening of its SMC hinge. *Cell* 127 523–537. 10.1016/j.cell.2006.08.048 17081975

[B38] Gruber S. Haering C. H. Nasmyth K. (2003). Chromosomal cohesin forms a ring. *Cell* 112 765–777. 12654244 10.1016/s0092-8674(03)00162-4

[B39] Guacci V. Koshland D. Strunnikov A. (1997). A direct link between sister chromatid cohesion and chromosome condensation revealed through the analysis of MCD1 in *S. cerevisiae*. *Cell* 91 47–57. 10.1016/S0092-8674(01)80008-8 9335334 PMC2670185

[B40] Haering C. H. Farcas A.-M. Arumugam P. Metson J. Nasmyth K. (2008). The cohesin ring concatenates sister DNA molecules. *Nature* 454 297–301. 10.1038/nature07098 18596691

[B41] Haering C. H. Löwe J. Hochwagen A. Nasmyth K. (2002). Molecular architecture of SMC proteins and the yeast cohesin complex. *Mol. Cell* 9 773–788. 10.1016/S1097-2765(02)00515-4 11983169

[B42] Hakimi M.-A. Bochar D. A. Schmiesing J. A. Dong Y. Barak O. G. Speicher D. W. (2002). A chromatin remodelling complex that loads cohesin onto human chromosomes. *Nature* 418 994–998. 10.1038/nature01024 12198550

[B43] Hauf S. Waizenegger I. C. Peters J. M. (2001). Cohesin cleavage by separase required for anaphase and cytokinesis in human cells. *Science* 293 1320–1323. 10.1126/science.1061376 11509732

[B44] Hirano T. (2006). At the heart of the chromosome: SMC proteins in action. *Nat. Rev. Mol. Cell Biol.* 7 311–322. 10.1038/nrm1909 16633335

[B45] Hirano T. (2012). Condensins: universal organizers of chromosomes with diverse functions. *Genes Dev.* 26 1659–1678. 10.1101/gad.194746.112 22855829 PMC3418584

[B46] Hou F. Zou H. (2005). Two human orthologues of Eco1/Ctf7 acetyltransferases are both required for proper sister-chromatid cohesion. *Mol. Biol. Cell* 16 3908–3918. 10.1091/mbc.E04 15958495 PMC1182326

[B47] Hu B. Itoh T. Mishra A. Katoh Y. Chan K. L. Upcher W. (2011). ATP hydrolysis is required for relocating cohesin from sites occupied by its Scc2/4 loading complex. *Curr. Biol.* 21 12–24. 10.1016/j.cub.2010.12.004 21185190 PMC4763544

[B48] Huang J. Hsu J. M. Laurent B. C. (2004). The RSC nucleosome-remodeling complex is required for cohesin’s association with chromosome arms. *Mol. Cell* 13 739–750. 15023343 10.1016/s1097-2765(04)00103-0

[B49] Jensen S. Segal M. Clarke D. J. Reed S. I. (2001). A novel role of the budding yeast separin Esp1 in anaphase spindle elongation: evidence that proper spindle association of Esp1 is regulated by Pds1. *J. Cell Biol.* 152 27–40. 10.1083/jcb.152.1.27 11149918 PMC2193664

[B50] Jiang L. Xia M. Strittmatter L. I. Makaroff C. A. (2007). The Arabidopsis cohesin protein SYN3 localizes to the nucleolus and is essential for gametogenesis. *Plant J.* 50 1020–1034. 10.1111/j.1365-313X.2007.03106.x 17488242

[B51] Jiang L. Yuan L. Xia M. Makaroff C. A. (2010). Proper levels of the Arabidopsis cohesion establishment factor CTF7 are essential for embryo and megagametophyte, but not endosperm, development. *Plant Physiol.* 154 820–832. 10.1104/pp.110.157560 20671110 PMC2949036

[B52] Kitajima T. S. Kawashima S. A. Watanabe Y. (2004). The conserved kinetochore protein shugoshin protects centromeric cohesion during meiosis. *Nature* 427 510–517. 10.1038/nature02312 14730319

[B53] Klebanow L. R. Peshel E. C. Schuster A. T. De K. Sarvepalli K. Lemieux M. E. (2016). Drosophila condensin II subunit, chromosome associated protein-D3, regulates cell fate determination through non-cell autonomous signaling. *Development* 143 2791–2802. 10.1242/dev.133686 27317808 PMC5004906

[B54] Klein F. Mahr P. Galova M. Buonomo S. B. C. Michaelis C. Nairz K. (1999). A central role for cohesins in sister chromatid cohesion, formation of axial elements, and recombination during yeast meiosis. *Cell* 98 91–103. 10.1016/S0092-8674(00)80609-1 10412984

[B55] Kueng S. Hegemann B. Peters B. H. Lipp J. J. Schleiffer A. Mechtler K. (2006). Wapl controls the dynamic association of cohesin with chromatin. *Cell* 127 955–967. 10.1016/j.cell.2006.09.040 17113138

[B56] Lam W. S. Yang X. Makaroff C. A. (2005). Characterization of *Arabidopsis thaliana* SMC1 and SMC3: evidence that AtSMC3 may function beyond chromosome cohesion. *J. Cell Sci.* 118 3037–3048. 10.1242/jcs.02443 15972315

[B57] Lengronne A. Katou Y. Mori S. Yokobayashi S. Kelly G. P. Itoh T. (2004). Cohesin relocation from sites of chromosomal loading to places of convergent transcription. *Nature* 430 573–578. 10.1038/nature02742 15229615 PMC2610358

[B58] Lengronne A. McIntyre J. Katou Y. Kanoh Y. Hopfner K. P. Shirahige K. (2006). Establishment of sister chromatid cohesion at the *S. cerevisiae* replication fork. *Mol. Cell* 23 787–799. 10.1016/j.molcel.2006.08.018 16962805

[B59] Lhuissier F. G. P. Offenberg H. H. Wittich P. E. Vischer N. O. E. Heyting C. (2007). The mismatch repair protein MLH1 marks a subset of strongly interfering crossovers in tomato. *Plant Cell* 19 862–876. 10.1105/tpc.106.049106 17337626 PMC1867368

[B60] Liu C. M. McElver J. Tzafrir I. Joosen R. Wittich P. Patton D. (2002). Condensin and cohesin knockouts in *Arabidopsis* exhibit a titan seed phenotype. *Plant J.* 29 405–415. 10.1046/j.1365-313x.2002.01224.x 11846874

[B61] Liu D. Makaroff C. A. (2015). Overexpression of a truncated CTF7 construct leads to pleiotropic defects in reproduction and vegetative growth in Arabidopsis. *BMC Plant Biol* 15:74 10.1186/s12870-015-0452-2 PMC4359560 25848842

[B62] Liu Z. Makaroff C. A. (2006). *Arabidopsis* separase AESP is essential for embryo development and the release of cohesin during meiosis. *Plant Cell* 18 1213–1225. 10.1105/tpc.105.036913 16582011 PMC1456861

[B63] Lopez-Serra L. Lengronne A. Borges V. Kelly G. Uhlmann F. (2013). Budding yeast Wapl controls sister chromatid cohesion maintenance and chromosome condensation. *Curr. Biol.* 23 64–69. 10.1016/j.cub.2012.11.030 23219725

[B64] Losada A. Hirano M. Hirano T. (2002). Cohesin release is required for sister chromatid resolution, but not for condensin-mediated compaction, at the onset of mitosis. *Genes Dev.* 16 3004–3016. 10.1101/gad.249202 12464631 PMC187494

[B65] Mayer M. L. Gygi S. P. Aebersold R. Hieter P. (2001). Identification of RFC (Ctf18p, Ctf8p, Dcc1p): an alternative RFC complex required for sister chromatid cohesion in *S. cerevisiae*. *Mol. Cell* 7 959–970. 10.1016/S1097-2765(01)00254-4 11389843

[B66] Mayer M. L. Pot I. Chang M. Xu H. Aneliunas V. Kwok T. (2004). Identification of protein complexes required for efficient sister chromatid cohesion. *Mol. Biol. Cell* 15 1736–1745. 10.1091/mbc.E03-08-0619 14742714 PMC379271

[B67] McAleenan A. Clemente-Blanco A. Cordon-Preciado V. Sen N. Esteras M. Jarmuz A. (2013). Post-replicative repair involves separase-dependent removal of the kleisin subunit of cohesin. *Nature* 493 250–254. 10.1038/nature11630 23178808

[B68] Mehta G. D. Kumar R. Srivastava S. Ghosh S. K. (2013). Cohesin: functions beyond sister chromatid cohesion. *FEBS Lett.* 587 2299–2312. 10.1016/j.febslet.2013.06.035 23831059

[B69] Mengiste T. Revenkova E. Bechtold N. Paszkowski J. (1999). An SMC-like protein is required for efficient homologous recombination in arabidopsis. *EMBO J.* 18 4505–4512. 10.1093/emboj/18.16.4505 10449416 PMC1171525

[B70] Mercier R. Armstrong S. Horlow C. Jackson N. Makaroff C. Vezon D. (2003). The meiotic protein SWI1 is required for axial element formation and recombination initiation in *Arabidopsis*. *Development* 130 3309–3318. 10.1242/dev.00550 12783800

[B71] Mercier R. Grelon M. Vezon D. Horlow C. Pelletier G. (2001). How to characterize meiotic functions in plants? *Biochimie* 83 1023–1028. 10.1016/S0300-9084(01)01348-7 11879730

[B72] Milutinovich M. Ünal E. Ward C. Skibbens R. V. Koshland D. (2007). A multi-step pathway for the establishment of sister chromatid cohesion. *PLoS Genet.* 3:e12 10.1371/journal.pgen.0030012 PMC1779304 17238288

[B73] Minina E. A. Reza S. H. Gutierrez-Beltran E. Elander P. H. Bozhkov P. V. Moschou P. N. (2017). Arabidopsis homologue of Scc4/MAU2 is essential for plant embryogenesis. *J. Cell Sci.* 130 1051–1063. 10.1242/jcs.196865 28137757

[B74] Moldovan G. L. Pfander B. Jentsch S. (2006). PCNA controls establishment of sister chromatid cohesion during S phase. *Mol. Cell* 23 723–732. 10.1016/j.molcel.2006.07.007 16934511

[B75] Musacchio A. Salmon E. D. (2007). The spindle-assembly checkpoint in space and time. *Nat. Rev. Mol. Cell Biol.* 8 379–393. 10.1038/nrm2163 17426725

[B76] Nasmyth K. (2000). Splitting the chromosome: cutting the ties that bind sister chromatids. *Science* 288 1379–1384. 10.1126/science.288.5470.1379 10827941

[B77] Nasmyth K. Haering C. H. (2009). Cohesin: its roles and mechanisms. *Annu. Rev. Genet.* 43 525–558. 10.1146/annurev-genet-102108-134233 19886810

[B78] Nishiyama T. Ladurner R. Schmitz J. Kreidl E. Schleiffer A. Bhaskara V. (2010). Sororin mediates sister chromatid cohesion by antagonizing Wapl. *Cell* 143 737–749. 10.1016/j.cell.2010.10.031 21111234

[B79] Nonaka N. Kitajima T. Yokobayashi S. Xiao G. Yamamoto M. Grewal S. I. S. (2002). Recruitment of cohesin to heterochromatic regions by Swi6/HP1 in fission yeast. *Nat. Cell Biol.* 4 89–93. 10.1038/ncb739 11780129

[B80] Onn I. Heidinger-Pauli J. M. Guacci V. Unal E. Koshland D. E. (2008). Sister chromatid cohesion: a simple concept with a complex reality. *Annu. Rev. Cell Dev. Biol.* 24 105–129. 10.1146/annurev.cellbio.24.110707.175350 18616427

[B81] Ono T. Losada A. Hirano M. Myers M. P. Neuwald A. F. Hirano T. (2003). Differential contributions of condensin I and condensin II to mitotic chromosome architecture in vertebrate cells. *Cell* 115 109–121. 10.1016/S0092-8674(03)00724-4 14532007

[B82] Ouyang Z. Zheng G. Song J. Borek D. M. Otwinowski Z. Brautigam C. A. (2013). Structure of the human cohesin inhibitor Wapl. *Proc. Natl. Acad. Sci. U.S.A.* 110 11355–11360. 10.1073/pnas.1304594110 23776203 PMC3710786

[B83] Palecek J. Vidot S. Feng M. Doherty A. J. Lehmann A. R. (2006). The Smc5-Smc6 DNA repair complex: bridging of the Smc5-Smc6 heads by the kleisin, nse4, and non-kleisin subunits. *J. Biol. Chem.* 281 36952–36959. 10.1074/jbc.M608004200 17005570

[B84] Pawlowski W. Wang C. Golubovskaya I. N. Szymaniak J. M. Shi L. Hamant O. (2009). Maize AMEIOTIC1 is essential for multiple early meiotic processes and likely required for the initiation of meiosis. *Proc. Natl. Acad. Sci. U.S.A.* 106 3603–3608. 10.1073/pnas.0810115106 19204280 PMC2637902

[B85] Pebernard S. McDonald H. W. Pavlova Y. Yates J. R. III Boddy M. N. (2004). Nse1, Nse2, and a novel subunit of the Smc5-Smc6 complex, Nse3, play a crucial role in meiosis. *Mol. Biol. Cell* 15 4866–4876. 10.1091/mbc.E04-05-6 15331764 PMC524734

[B86] Peirson B. N. Bowling S. E. Makaroff C. A. (1997). A defect in synapsis causes male sterility in a T-DNA-tagged *Arabidopsis thaliana* mutant. *Plant J.* 11 659–669. 10.1046/j.1365-313X.1997.11040659.x 9161029

[B87] Peters J.-M. Nishiyama T. (2012). Sister chromatid cohesion. *Cold Spring Harb. Perspect. Biol.* 4:a011130 10.1101/cshperspect.a011130 PMC3536341 23043155

[B88] Petronczki M. Chwalla B. Siomos M. F. Yokobayashi S. Helmhart W. Deutschbauer A. M. (2004). Sister-chromatid cohesion mediated by the alternative RF-CCtf18/Dcc1/Ctf8, the helicase Chl1 and the polymerase-alpha-associated protein Ctf4 is essential for chromatid disjunction during meiosis II. *J. Cell Sci.* 117 3547–3559. 10.1242/jcs.01231 15226378

[B89] Pradillo M. Knoll A. Oliver C. Varas J. Corredor E. Puchta H. (2015). Involvement of the cohesin cofactor PDS5 (SPO76) during meiosis and DNA repair in *Arabidopsis thaliana*. *Front. Plant Sci.* 6:1034 10.3389/fpls.2015.01034 PMC4664637 26648949

[B90] Prieto I. Suja J. A. Pezzi N. Kremer L. Martínez-A C. Rufas J. S. (2001). Mammalian STAG3 is a cohesin specific to sister chromatid arms in meiosis I. *Nat. Cell Biol.* 3 761–766. 10.1038/35087082 11483963

[B91] Qi Y. Zhang Y. Zhang F. Baller J. A. Cleland S. C. Ryu Y. (2013). Increasing frequencies of site-specific mutagenesis and gene targeting in Arabidopsis by manipulating DNA repair pathways. *Genome Res.* 23 547–554. 10.1101/gr.145557.112 23282329 PMC3589543

[B92] Rankin S. Ayad N. G. Kirschner M. W. (2005). Sororin, a substrate of the anaphase- promoting complex, is required for sister chromatid cohesion in vertebrates. *Mol. Cell* 18 185–200. 10.1016/j.molcel.2005.03.017 15837422

[B93] Revenkova E. Eijpe M. Heyting C. Gross B. Jessberger R. (2001). Novel meiosis-specific isoform of mammalian SMC1. *Mol. Cell. Biol.* 21 6984–6998. 10.1128/MCB.21.20.6984-6998.2001 11564881 PMC99874

[B94] Ritchie K. Seah C. Moulin J. Isaac C. Dick F. Bérubé N. G. (2008). Loss of ATRX leads to chromosome cohesion and congression defects. *J. Cell Biol.* 180 315–324. 10.1083/jcb.200706083 18227278 PMC2213576

[B95] Rivera T. Losada A. (2009). Shugoshin regulates cohesion by driving relocalization of PP2A in *Xenopus* extracts. *Chromosoma* 118 223–233. 10.1007/s00412-008-0190-4 18987869

[B96] Rivera T. Losada A. (2010). Recycling cohesin rings by deacetylation. *Mol. Cell* 39 657–659. 10.1016/j.molcel.2010.08.032 20832715

[B97] Rolef Ben-Shahar T. Heeger S. Lehane C. East P. Flynn H. Skehel M. (2008). Eco1-dependent cohesin acetylation during establishment of sister chromatid cohesion. *Science* 321 563–566. 10.1126/science.1157774 18653893

[B98] Rowland B. D. Roig M. B. Nishino T. Kurze A. Uluocak P. Mishra A. (2009). Building sister chromatid cohesion: Smc3 acetylation counteracts an antiestablishment activity. *Mol. Cell* 33 763–774. 10.1016/j.molcel.2009.02.028 19328069

[B99] Sakamoto T. Inui Y. T. Uraguchi S. Yoshizumi T. Matsunaga S. Mastui M. (2011). Condensin II alleviates DNA damage and is essential for tolerance of boron overload stress in *Arabidopsis*. *Plant Cell* 23 3533–3546. 10.1105/tpc.111.086314 21917552 PMC3203421

[B100] Schmidt C. K. Brookes N. Uhlmann F. (2009). Conserved features of cohesin binding along fission yeast chromosomes. *Genome Biol.* 10:R52 10.1186/gb-2009-10-5-r52 PMC2718518 19454013

[B101] Schmitz J. Watrin E. Lénárt P. Mechtler K. Peters J. M. (2007). Sororin is required for stable binding of cohesin to chromatin and for sister chromatid cohesion in interphase. *Curr. Biol.* 17 630–636. 10.1016/j.cub.2007.02.029 17349791

[B102] Schubert V. (2009). SMC proteins and their multiple functions in higher plants. *Cytogenet. Genome Res.* 124 202–214. 10.1159/000218126 19556774

[B103] Schubert V. Lermontova I. Schubert I. (2013). The *Arabidopsis* CAP-D proteins are required for correct chromatin organisation, growth and fertility. *Chromosoma* 122 517–533. 10.1007/s00412-013-0424-y 23929493

[B104] Sebastian J. Ravi M. Andreuzza S. Panoli A. P. Marimuthu M. P. A. Siddiqi I. (2009). The plant adherin AtSCC2 is required for embryogenesis and sister-chromatid cohesion during meiosis in Arabidopsis. *Plant J.* 59 1–13. 10.1111/j.1365-313X.2009.03845.x 19228337

[B105] Seitan V. C. Banks P. Laval S. Majid N. A. Dorsett D. Rana A. (2006). Metazoan Scc4 homologs link sister chromatid cohesion to cell and axon migration guidance. *PLoS Biol.* 4:e242 10.1371/journal.pbio.0040242 PMC1484498 16802858

[B106] Singh D. K. Andreuzza S. Panoli A. P. Siddiqi I. (2013). AtCTF7 is required for establishment of sister chromatid cohesion and association of cohesin with chromatin during meiosis in *Arabidopsis*. *BMC Plant Biol.* 13:117 10.1186/1471-2229-13-7 PMC3751900 23941555

[B107] Sjögren C. Nasmyth K. (2001). Sister chromatid cohesion is required for postreplicative double-strand break repair in *Saccharomyces cerevisiae*. *Curr. Biol.* 11 991–995. 10.1016/S0960-9822(01)00271-8 11448778

[B108] Skibbens R. V. Corson L. B. Koshland D. Hieter P. (1999). Ctf7p is essential for sister chromatid cohesion and links mitotic chromosome structure to the DNA replication machinery. *Genes Dev.* 13 307–319. 10.1101/gad.13.3.307 9990855 PMC316428

[B109] Stemmann O. Zou H. Gerber S. A. Gygi S. P. Kirschner M. W. (2001). Dual inhibition of sister chromatid separation at metaphase. *Cell* 107 715–726. 10.1016/S0092-8674(01)00603-1 11747808

[B110] Storlazzi A. Tesse S. Ruprich-Robert G. Gargano S. Pöggeler S. Kleckner N. (2008). Coupling meiotic chromosome axis integrity to recombination. *Genes Dev.* 22 796–809. 10.1101/gad.459308 18347098 PMC2275432

[B111] Ström L. Karlsson C. Lindroos H. B. Wedahl S. Katou Y. Shirahige K. (2007). Postreplicative formation of cohesion is required for repair and induced by a single DNA break. *Science* 317 242–245. 10.1126/science.1140649 17626884

[B112] Sumara I. Vorlaufer E. Gieffers C. Peters B. H. Peters J. M. (2000). Characterization of vertebrate cohesin complexes and their regulation in prophase. *J. Cell Biol.* 151 749–761. 10.1083/jcb.151.4.749 11076961 PMC2169443

[B113] Sutani T. Kawaguchi T. Kanno R. Itoh T. Shirahige K. (2009). Budding yeast Wpl1(Rad61)-Pds5 complex counteracts sister chromatid cohesion-establishing reaction. *Curr. Biol.* 19 492–497. 10.1016/j.cub.2009.01.062 19268589

[B114] Takahashi T. S. Basu A. Bermudez V. Hurwitz J. Walter J. C. (2008). Cdc7-Drf1 kinase links chromosome cohesion to the initiation of DNA replication in *Xenopus* egg extracts. *Genes Dev.* 22 1894–1905. 10.1101/gad.1683308 18628396 PMC2492736

[B115] Takahashi T. S. Yiu P. Chou M. F. Gygi S. Walter J. C. (2004). Recruitment of *Xenopus* Scc2 and cohesin to chromatin requires the pre-replication complex. *Nat. Cell Biol.* 6 991–996. 10.1038/ncb1177 15448702

[B116] Tang Z. Shu H. Qi W. Mahmood N. A. Mumby M. C. Yu H. (2006). PP2A Is required for centromeric localization of Sgo1 and proper chromosome segregation. *Dev. Cell* 10 575–585. 10.1016/j.devcel.2006.03.010 16580887

[B117] Tapia-Alveal C. Lin S.-J. O’Connell M. J. (2014). Functional interplay between cohesin and Smc5/6 complexes. *Chromosoma* 123 437–445. 10.1007/s00412-014-0474-9 24981336 PMC4169997

[B118] Tomonaga T. Nagao K. Kawasaki Y. Furuya K. Murakaini A. Morishita J. (2000). Characterization of fission yeast cohesin: essential anaphase proteolysis of Rad21 phosphorylated in the S phase. *Genes Dev.* 14 2757–2770. 10.1101/gad.832000 11069892 PMC317025

[B119] Tóth A. Ciosk R. Uhlmann F. Galova M. Schleiffer A. Nasmyth K. (1999). Yeast cohesin complex requires a conserved protein, Eco1p(Ctf7), to establish cohesion between sister chromatids during DNA replication. *Genes Dev.* 13 320–333. 10.1101/gad.13.3.320 9990856 PMC316435

[B120] Toyoda Y. Furuya K. Goshima G. Nagao K. Takahashi K. Yanagida M. (2002). Requirement of chromatid cohesion proteins Rad21/Scc1 and Mis4/Scc2 for normal spindle-kinetochore interaction in fission yeast. *Curr. Biol.* 12 347–358. 10.1016/S0960-9822(02)00692-9 11882285

[B121] Tsubouchi T. Roeder G. S. (2005). A synaptonemal complex protein promotes homology-independent centromere coupling. *Science* 308 870–873. 10.1126/science.1108283 15879219

[B122] Uhlmann F. Lottspeich F. Nasmyth K. (1999). Sister-chromatid separation at anaphase onset is promoted by cleavage of the cohesin subunit Scc1. *Nature* 400 37–42. 10.1038/21831 10403247

[B123] Uhlmann F. Nasmyth K. (1998). Cohesion between sister chromatids must be established during DNA replication. *Curr. Biol.* 8 1095–1101. 10.1016/S0960-9822(98)70463-4 9778527

[B124] Unal E. Heidinger-Pauli J. M. Kim W. Guacci V. Onn I. Gygi S. P. (2008). A molecular determinant for the establishment of sister chromatid cohesion. *Science* 321 566–569. 10.1126/science.1157880 18653894

[B125] Vaur S. Feytout A. Vazquez S. Javerzat J. (2012). Pds5 promotes cohesin acetylation and stable cohesin–chromosome interaction. *EMBO Rep.* 13 645–652. 10.1038/embor.2012.72 22640989 PMC3388792

[B126] Vernì F. Gandhi R. Goldberg M. L. Gatti M. (2000). Genetic and molecular analysis of wings apart-like (wapl), a gene controlling heterochromatin organization in *Drosophila melanogaster*. *Genetics* 154 1693–1710. 10747063 10.1093/genetics/154.4.1693 PMC1461031

[B127] Verver D. Zheng Y. Speijer D. Hoebe R. Dekker H. L. Repping S. (2016). Non-SMC element 2 (NSMCE2) of the SMC5/6 complex helps to resolve topological stress. *Int. J. Mol. Sci.* 17:1782 10.3390/ijms17111782 PMC5133783 27792189

[B128] Waizenegger I. C. Hauf S. Meinke A. Peters J. M. (2000). Two distinct pathways remove mammalian cohesin from chromosome arms in prophase and from centromeres in anaphase. *Cell* 103 399–410. 10.1016/S0092-8674(00)00132-X 11081627

[B129] Warren C. D. Eckley D. M. Lee M. S. Hanna J. S. Hughes A. Peyser B. (2004). S-phase checkpoint genes safeguard high-fidelity sister chromatid cohesion. *Mol. Biol. Cell* 15 1724–1735. 10.1091/mbc.E03-09-7 14742710 PMC379270

[B130] Watanabe K. Pacher M. Dukowic S. Schubert V. Puchta H. Schubert I. (2009). The STRUCTURAL MAINTENANCE OF CHROMOSOMES 5/6 complex promotes sister chromatid alignment and homologous recombination after DNA damage in *Arabidopsis thaliana*. *Plant Cell* 21 2688–2699. 10.1105/tpc.108.060525 19737979 PMC2768936

[B131] Watanabe Y. Nurse P. (1999). Cohesin Rec8 is required for reductional chromosome segregation at meiosis. *Nature* 400 461–464. 10.1038/22774 10440376

[B132] Watrin E. Schleiffer A. Tanaka K. Eisenhaber F. Nasmyth K. Peters J. M. (2006). Human Scc4 is required for cohesin binding to chromatin, sister-chromatid cohesion, and mitotic progression. *Curr. Biol.* 16 863–874. 10.1016/j.cub.2006.03.049 16682347

[B133] Weber S. A. Gerton J. L. Polancic J. E. DeRisi J. L. Koshland D. Megee P. C. (2004). The kinetochore is an enhancer of pericentric cohesin binding. *PLoS Biol.* 2:E260 10.1371/journal.pbio.0020260 PMC490027 15309047

[B134] Woo J. S. Lim J. H. Shin H. C. Suh M. K. Ku B. Lee K. H. (2009). Structural studies of a bacterial condensin complex reveal ATP-dependent disruption of intersubunit interactions. *Cell* 136 85–96. 10.1016/j.cell.2008.10.050 19135891

[B135] Wu N. Kong X. Ji Z. Zeng W. Potts P. R. Yokomori K. (2012). Scc1 sumoylation by Mms21 promotes sister chromatid recombination through counteracting Wapl. *Genes Dev.* 26 1473–1485. 10.1101/gad.193615.112 22751501 PMC3403015

[B136] Wu S. Scheible W.-R. Schindelasch D. Van Den Daele H. De Veylder L. Baskin T. I. (2010). A conditional mutation in *Arabidopsis thaliana* separase induces chromosome non-disjunction, aberrant morphogenesis and cyclin B1;1 stability. *Development* 137 953–961. 10.1242/dev.041939 20150278

[B137] Xu P. Yuan D. Liu M. Li C. Liu Y. Zhang S. (2013). AtMMS21, an SMC5/6 complex subunit, is involved in stem cell niche maintenance and DNA damage responses in Arabidopsis roots. *Plant Physiol.* 161 1755–1768. 10.1104/pp.112.208942 23426194 PMC3613453

[B138] Yang X. Boateng K. A. Strittmatter L. Burgess R. Makaroff C. A. (2009). Arabidopsis separase functions beyond the removal of sister chromatid cohesion during meiosis. *Plant Physiol.* 151 323–333. 10.1104/pp.109.140699 19592426 PMC2735979

[B139] Yuan L. Yang X. Makaroff C. A. (2011). Plant cohesins, common themes and unique roles. *Curr. Protein Pept. Sci.* 12 93–104. 10.2174/138920311795684904 21348848

[B140] Zamariola L. De Storme N. Tiang C. L. Armstrong S. J. Franklin F. C. H. Geelen D. (2013). SGO1 but not SGO2 is required for maintenance of centromere cohesion in *Arabidopsis thaliana* meiosis. *Plant Reprod.* 26 197–208. 10.1007/s00497-013-0231-x 23884434

[B141] Zamariola L. Tiang C. L. De Storme N. Pawlowski W. Geelen D. (2014). Chromosome segregation in plant meiosis. *Front. Plant Sci.* 5:279 10.3389/fpls.2014.00279 PMC4060054 24987397

[B142] Zhang J. Shi X. Li Y. Kim B. J. Jia J. Huang Z. (2008). Acetylation of Smc3 by Eco1 is required for S phase sister chromatid cohesion in both human and yeast. *Mol. Cell* 31 143–151. 10.1016/j.molcel.2008.06.006 18614053

[B143] Zhang L. Tao J. Wang S. Chong K. Wang T. (2006). The rice OsRad21-4, an orthologue of yeast Rec8 protein, is required for efficient meiosis. *Plant Mol. Biol.* 60 533–554. 10.1007/s11103-005-4922-z 16525890

[B144] Zhao D. Yang X. Quan L. Timofejeva L. Rigel N. W. Ma H. (2006). ASK1, a SKP1 homolog, is required for nuclear reorganization, presynaptic homolog juxtaposition and the proper distribution of cohesin during meiosis in Arabidopsis. *Plant Mol. Biol.* 62 99–110. 10.1007/s11103-006-9006-1 16897472

